# Unmet Supportive Care Needs Among Women With Breast and Gynecological Cancer: Relevance of Attachment Anxiety and Psychological Distress

**DOI:** 10.3389/fpsyg.2020.558190

**Published:** 2020-10-21

**Authors:** Johanna Graf, Florian Junne, Johannes C. Ehrenthal, Norbert Schäffeler, Juliane Schwille-Kiuntke, Andreas Stengel, Anja Mehnert-Theuerkauf, Lennart Marwedel, Sara Y. Brucker, Stephan Zipfel, Martin Teufel

**Affiliations:** ^1^Department of Psychosomatic Medicine and Psychotherapy, University Hospital Tuebingen, Tuebingen, Germany; ^2^Comprehensive Cancer Center, University Hospital Tuebingen, Tuebingen, Germany; ^3^Institute of Medical Psychology, Heidelberg University, Heidelberg, Germany; ^4^Institute of Occupational and Social Medicine and Health Services Research, University of Tuebingen, Tuebingen, Germany; ^5^Charité Center for Internal Medicine and Dermatology, Department for Psychosomatic Medicine, Charité-Universitätsmedizin Berlin, Corporate Member of Freie Universität Berlin, Humboldt-Universität zu Berlin, Berlin Institute of Health, Berlin, Germany; ^6^Department of Medical Psychology and Medical Sociology, University Medical Center Leipzig, Leipzig, Germany; ^7^Department of Obstetrics and Gynecology, University of Tuebingen, Tuebingen, Germany; ^8^Department of Psychosomatic Medicine and Psychotherapy, LVR University Hospital, Essen, University of Duisburg-Essen, Essen, Germany

**Keywords:** attachment styles, attachment anxiety, distress, psychooncology, supportive care needs, unmet needs

## Abstract

**Objective:**

Attachment anxiety and avoidance are known risk factors for the development of unmet needs and poor well-being among patients with chronic diseases. Few studies have addressed this in individuals with cancer. We aimed to explore the relationship between supportive care needs, attachment styles and distress in women with breast and gynecological cancer.

**Methods:**

Using a cross-sectional paper-pencil (*n* = 157) and online survey (*n* = 614), a total of 771 patients with breast or gynecological cancer completed a set of validated questionnaires. From September 2013 to January 2014, consecutive inpatients and outpatients of the university hospital Tuebingen were included in the study. Further, participants were recruited through social media, patient internet platforms, self-help group leaders and patient networks. We used the Supportive Care Needs Survey (SCNS-SF-34) with the need dimensions: health system, patient care, psychological, physical, and sexual needs, as well as the Experiences in Close Relationships-Revised Questionnaire, and the Distress Thermometer. A multiple linear regression model was used to analyze the influence of attachment styles (anxiety and avoidance) on the SCNS-SF-34 dimensions. A moderation analysis was used to explore the influence of the interaction between attachment anxiety and distress for all SCNS-SF-34 dimensions.

**Results:**

Attachment anxiety was a significant determinant and led to higher unmet supportive care needs in all dimensions, whereas attachment avoidance was not significant. Distress did moderate the relationship between attachment anxiety and psychological and health system needs and led to a higher unmet needs development. For the other SCNS-SF-34 dimensions, distress was not confirmed as a moderator.

**Conclusion:**

Our findings highlight attachment anxiety as a risk factor for the development of unmet supportive care needs and potentially impaired psychological adjustment to cancer. Further studies are needed to elucidate the interactions between attachment styles, distress and supportive care needs among cancer patients.

## Introduction

The diagnosis of breast or gynecological cancer, along with the long-term invasive treatments like chemotherapy, radiotherapy, and/or surgery can lead to various psychological morbidities. The affected person can feel sad, threatened, and uncertain (Dunn and Steginga, 2000; Ahmad et al., 2015) leading to the development of high cancer-related distress (Zabora et al., 2001) and/or clinically relevant symptoms (e.g., adjustment disorder, anxiety, and depression) (Mehnert et al., 2014; Mielcarek et al., 2016). In relation to their high disease-related distress, patients – especially women with breast and gynecological cancer – can experience unmet supportive care needs during their illness (Schmid-Büchi et al., 2008; Roland et al., 2013). Unmet supportive care needs are defined as a lack of service or support that an individual perceives as necessary to reach the best possible well-being (Fitch, 2000). Younger patients, women, patients with a hereditary cancer risk or with high anxious or depressive symptoms, and patients living alone express more unmet supportive care needs and are at higher risk of poor adjustment to a cancer diagnosis and have reduced ability to cope with the demands of the disease (Ahmad et al., 2015; Faller et al., 2015; Jeppesen et al., 2015; Brédart et al., 2016; Ringwald et al., 2016). However, little is known about general trait factors associated with high levels of perceived unmet supportive care needs and poor well-being, and there is a lack of evidence regarding personality factors associated with the development of unmet supportive care needs (Faller et al., 2016).

In recent years, researchers and clinicians have begun to focus more on attachment theory as a framework for understanding adjustment to illness and disease (Nicholls et al., 2014; Nissen, 2016). Attachment theory describes the development and dynamics of relatively stable social-cognitive schemes (“internal working models”), which organize the processing of attachment-related information, influence self- and interpersonal stress regulation and guide-related behavior over the lifespan. The attachment system is activated in times of need or distress and aims at restoring a subjective sense of security (Bowlby, 1969; Mikulincer and Shaver, 2007; Ainsworth et al., 2014). Attachment in adulthood can be described by central patterns of perception, motivation, regulation, and behavior, often called “attachment styles” (Mikulincer et al., 2003; Ainsworth et al., 2014).

Individuals with a prototypically secure attachment style are confident that others will be there for them in times of need and therefore feel comfortable in seeking and receiving the help of others, but are also able to self-regulate due to the activation of self-soothing memories of the generally positive caregiving history (Mikulincer et al., 2003).

Attachment insecurity (i.e., concerning the question if others are there in times of distress) is often described in terms of attachment anxiety or attachment avoidance (Ainsworth et al., 2014). Attachment anxiety describes the attempt to adapt to this insecurity by the hyperactivation of attachment-related emotions, cognitions, and behavior, while habitually neglecting self-regulatory strategies. In particular, there is an increased fear of rejection or abandonment and heightened levels of distress when potential caregivers are unavailable or unresponsive, accompanied by increased care-seeking and interpersonal dependency (Mikulincer et al., 2003; Ainsworth et al., 2014). Attachment avoidance describes the attempt to respond to the general insecurity by downregulating attachment-related emotions, cognitions and behavior, while neglecting others’ regulatory competence. Attachment avoidance often leads to a devaluation of close relationships, increased interpersonal distance, excessive focus on self-reliance and reluctance to self-disclose (Mikulincer et al., 2003; Ainsworth et al., 2014). It is important to keep in mind that insecure attachment styles are normal variants of different developmental trajectories (Mikulincer and Shaver, 2007). However, insecure attachment regulatory styles can be considered as risk factors for maladaptive behaviors when habitual attachment-related mechanisms no longer match the regulatory task, especially in the face of stress and strain. In other words, the adaptiveness of both high attachment anxiety and avoidance may break down under certain conditions (Gillath et al., 2009), influencing the perception and interpersonal modulation of stress, the psychobiological stress response, self-regulation, and health behavior, ultimately affecting health-related outcomes (Maunder et al., 2015).

The mechanism of attachment theory, often neglected in medical research, is crucial for understanding its potential impact on health behavior and disease development. Bowlby suggested that physical illness is likely to activate the attachment behavioral system due to experienced distress, unmet needs and perceived vulnerability (Bowlby, 1969). Studies in the context of chronic diseases such as cancer have demonstrated that attachment styles can predict psychological adjustment and well-being (Schmidt et al., 2002; Turner-Cobb et al., 2002; Hamama-Raz and Solomon, 2006; Porter et al., 2012; Vehling et al., 2019). It has been shown that attachment anxiety leads to higher psychological distress and increased levels of endocrine stress responses (Ehrenthal et al., 2011; Arambasic et al., 2019). Individuals with higher levels of attachment avoidance usually report lower levels of psychological burden than individuals with higher scores of attachment anxiety (Dozier and Lee, 1995). In patients with cancer, insecurely attached individuals use less active and less positive coping strategies to manage their diagnosis of cancer and survivorship issues, such as physical and emotional consequences of the cancer treatment (Schmidt et al., 2012; Arambasic et al., 2019; Romeo et al., 2019). Moreover, related studies have shown that attachment anxiety is associated with depression (Hunter et al., 2006; Porter et al., 2012; Nissen, 2016; Scheffold et al., 2017), higher symptoms of anxiety and reduced social well-being and quality of life among cancer patients (Porter et al., 2012; Smith et al., 2018; Arambasic et al., 2019; Romeo et al., 2019). This is of special relevance as the effect of attachment anxiety on health-related outcomes (e.g., medical symptoms, overall health and bodily pain) may be moderated by the perception of social support (Stanton and Campbell, 2014; McWilliams, 2017). Insecure attachment, at the same time, is associated with lower levels of social support (Hunter et al., 2006; Nissen, 2016). Attachment security and the perceived security about the availability of others can protect from demoralization. Further, low attachment security may limit adaptive capacity to deal with illness burden and discourage morale and purpose in life with advanced cancer (Vehling et al., 2019).

However, it remains unclear how attachment insecurity and distress determine the perception of unmet supportive care needs. In previous research it was shown that distress also leads to an increased development of unmet supportive care needs in patients with cancer (Faller et al., 2017). However, it is not clear if perceived distress moderates the relationship between attachment styles and supportive care needs (that is, attachment insecurity only impacts unfulfilled supportive care needs if distress is also present) or attachment insecurity directly impacts both distress and unmet supportive care needs (van Scheppingen et al., 2011). Systematic reviews urgently call for further research focusing on attachment styles to better understand apparent inconsistencies in research into the interactions between supportive care needs and the well-being of cancer patients (Nicholls et al., 2014; Nissen, 2016).

**FIGURE 1:**
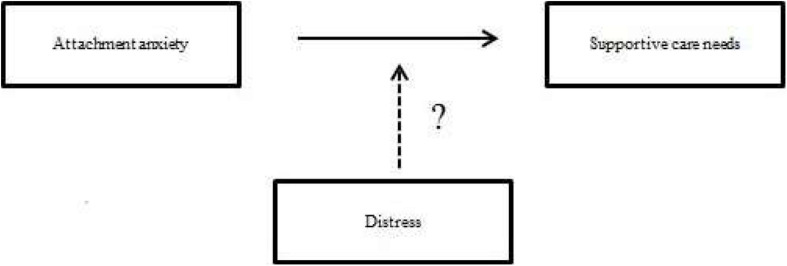
The possible moderation of distress on the development of supportive care needs.

Given the evidence gap on the pressing issues described above, the current study focuses on two key goals: (1) to define the relationship between insecure attachment styles (anxiety and avoidance) and perceived supportive care needs, and (2) to investigate the moderation effect of perceived distress on the relationship between attachment styles (anxiety and avoidance) and supportive care needs in women with breast and gynecological cancer (Figure 1).

## Materials and Methods

### Study Design and Recruitment

In a cross-sectional approach 1172 women with either breast or gynecological cancer or both *agreed to participate in the study*. Eligibility criteria were defined as being an adult (age ≥18 years) and having sufficient German language skills to complete a set of questionnaires. The survey data were collected *via* self-report paper-pencil or self-report online questionnaires and took approximately 20 min to complete. From September 2013 to January 2014, consecutive inpatients and outpatients were approached regarding participation (paper-pencil-questionnaire at Department of Gynecology at the University Hospital Tuebingen). The research assistants asked inpatients and outpatients; after a time of consideration the patients could decide to fill in the paper-pencil or online version of the questionnaires. Patients could choose to send the paper-pencil questionnaires back with the attached envelope or hand it back immediately to the research assistants. Furthermore, breast and gynecological cancer patients were recruited *via* an electronic online survey version (Questback) of the questionnaire through social media, special patient internet platforms, self-help group leaders, patient networks (e.g., Breast Cancer Aid Germany; BRCA Network) and further cancer counseling centers. The survey was anonymous and the beginning of the questionnaire was the consent page. An incentive was not given. Of the 1172 participants (*n* = 243 paper-pencil and *n* = 929 online) assessed, 41 patients assessed online did not meet the eligibility criteria because of another cancer diagnosis. Those with incomplete data (*n* = 360) were excluded, resulting in a final dataset of 771 participants, of which 614 were completed online and 157 as paper-pencil questionnaires.

### Procedures

The local ethics committee of the University Hospital Tuebingen approved the study protocol.

### Measures

#### Demographic and Disease-Related Information

Demographic variables included age, gender, marital status, number, and age of children. Self-reported data on the type of cancer, time since primary diagnosis, and disease status (primary disease, metastasis, and recurrence) was also collected.

#### Supportive Care Needs Survey

The Supportive Care Needs Survey is a 34-item short-form version (SCNS-SF-34). We used the German version of SCNS-SF-34, which has good psychometric properties (Lehmann et al., 2012). This self-report questionnaire assesses patients’ perceived type and extent of need for support in five dimensions: (1) *health system/information needs*; (2) *patient care and support needs*, (3) *psychological needs*; (4) *physical and daily living needs*, and (5) *sexual needs*. Example items are “*In the last month what was your level of need with learning to feel in control of your situation?”* or “*In the last month what was your level of need with feeling down or depressed?”.* The patient ranks their needs on a five-point Likert scale, ranging from 1 to 5 (1 = no need; 2 = no need; satisfied; 3 = low need; 4 = moderate need; and 5 = high need). Summated scores for the five dimensions were first calculated and converted to scores ranging from 0 to 100 for each domain. Standardized scores were then calculated, in which higher scores indicate unmet supportive care needs within that domain.

#### Experiences in Close Relationships–Revised Questionnaire

Attachment styles were measured using a brief German version of the Experiences in Close Relationships-Revised Questionnaire (ECR-RD) (Fraley et al., 2000; Ehrenthal et al., 2009). The ECR-RD assesses experiences and expectations regarding romantic relationships on two scales of attachment-related anxiety (“I often worry that my partner does not *really* love me”) and avoidance (“I feel uncomfortable opening up to my partner”) on a seven-point Likert-scale, ranging from 1 (“strongly disagree”) to 7 (“strongly agree”). The brief version (ECR-RD8) was developed as a screening instrument suitable for large samples in health psychology and psychosomatic medicine. Using data from several published studies on the original 36-item version, a total of eight items were extracted by means of exploratory and confirmatory factor analysis. Four items belong to the dimension “attachment-related anxiety” and the remaining four items belong to the “attachment-related avoidance” dimension. The questionnaire was furthermore evaluated in a representative sample of the German population. Its internal consistency values are good, the model fit of the confirmatory factor analysis good to acceptable, and validity was established by comparing it to measures of psychological health as well as another attachment measure (Ehrenthal et al., in preparation). The long version of the ECR-RD8 RD has been, and the short version is currently used in a wide range of studies (Ohlsson, 2013; Manes et al., 2016; Ehrenthal et al., in preparation).

#### Distress Thermometer

The 11-level visual analog scale of the “Distress Thermometer” (DT) is widely used to measure distress and has been validated in diverse oncology settings (Mehnert et al., 2006). Patients were instructed to “choose a number indicating how much distress they have been feeling over the past week, including today. Zero means no distress and 10 means the worst distress imaginable.” A cut-off score ≥5 is recommended as indicative of a high distress level (Mehnert et al., 2006).

### Data Analysis

Descriptive statistics, correlations, and regression analyses were performed using SPSS 21 for Windows. First, multiple linear regression models were used to explore the possible influence of attachment styles (anxiety, avoidance) on the five need dimensions of the SNCS-SF-34. The correlation matrices are shown in Tables 1, 2. Due to the explorative character of our research we did not adjust the alpha-level. In a second step, a moderator analysis was conducted using the logistic path analytic model (model 1) using the SPSS PROCESS macro (Version 3.5). This moderator analysis was used to estimate the interaction between distress and attachment anxiety and their influence on the five need dimensions of the SCNS-SF-34. Lower level confidence intervals (LLCI) and upper level confidence intervals (ULCI) were calculated (Hayes, 2013). Within our models, the five need dimensions of the SCNS-SF-34 were the dependent variables, and attachment style (anxiety, avoidance), distress, and the interaction term (attachment anxiety × distress) were the independent variables (O’brien, 2007). Multicollinearity between determinants (attachment anxiety and distress) and the interaction term (attachment anxiety × distress) was prevented by using the centered scores of the component variables. Demographic variables were described using percentages and means as appropriate. Missing data were analyzed and mean missing values estimated as 8.9% for the SCNS-SF-34 questionnaire and 2.1% for the ECR-RD8 questionnaire. Missing values were imputed only if at least 80% of each questionnaire had been completed. Using the Little’s MCAR test, it was confirmed that the data were missing randomly. Therefore, missing data were imputed with the expectation-maximization (EM) algorithm (Musil et al., 2002). For all statistical tests, the level of significance was set to alpha at 0.05.

**TABLE 1:** Mean, standard deviations, and correlation with attachment anxiety.

**Variable**	**M**	**SD**	**1**	**2**	**3**	**4**	**5**
1. Attachment anxiety	13.85	8.55					
2. Health system/information needs	20.51	14.10	0.288*				
3. Patient care and support needs	7.09	6.37	0.285*	0.847*			
4. Psychological needs	20.52	11.85	0.333*	0.668*	0.653*		
5. Physical and daily living needs	8.04	5.38	0.225*	0.561*	0.603*	0.674*	
6. Sexual needs	5.49	3.87	0.386*	0.751*	0.686*	0.701*	0.591*

*M and SD are used to represent mean and standard deviation, respectively. ^∗^indicates *p* < 0.01*

**TABLE 2:** Mean, standard deviations, and correlation with attachment avoidance.

**Variable**	**M**	**SD**	**1**	**2**	**3**	**4**	**5**
1. Attachment avoidance	22.66	7.90					
2. Health system/information needs	20.51	14.10	0.046				
3. Patient care and support needs	7.09	6.37	−0.014	0.847*			
4. Psychological needs	20.52	11.85	0.000	0.668*	0.653*		
5. Physical and daily living needs	8.04	5.38	0.001	0.561*	0.603*	0.674*	
6. Sexual needs	5.49	3.87	0.021	0.701*	0.751*	0.686*	0.591*

*M and SD are used to represent mean and standard deviation, respectively. ^∗^indicates *p* < 0.01*

## Results

Our final sample consisted of 771 women. The mean patient age was 50.6 ± 10.5 years (range: 25–83 years). Seventy-six percent of the sample was diagnosed with cancer for the first time and 8.3% of participants were affected by metastases. A recurrence of a previous cancer affected 9.2% of the sample. 6.1 % of patients suffered from metastases and recurrence. The frequencies of other disease-related or demographic variables are provided in Table 3. The mean values and standard deviations of the SCNS-SF-34, ECR-RD8, and DT are presented in Table 4. In the sample, significant differences in demographic variables and distress exist between the paper-pencil and online groups. The Cohen’s effect size for the paper-pencil versus online comparison was less than 0.3; therefore, we assume that these differences were not clinically relevant (data not shown).

**TABLE 3:** Study population characteristics: sociodemographics and disease-related information.

***N* = 771**	**Mean (SD)**	**No. (%)**
Age (years)	50.6 (10.5) Range:25–83 years	
Length of time since first diagnosis and questionnaire completion	4.6 (5.1) Range:0–39 years	
Cancer diagnosis		
Breast		671 (87.0)
Gynecological		72 (9.3)
…Two cancer diagnosis		28 (3.7)
Disease status		
First		589 (76.4)
Metastasis		64 (8.3)
Reoccurrence		71 (9.2)
…Metastasis and Reoccurrence		47 (6.1)
Married/with a partner		
Yes		645 (83.7)
No		126 (16.3)
Children		
None		170 (22.0
Having children		598 (78.0))
1 child		176 (29.4)
2 children		263 (44.0)
3 children		103 (17.2)
4 children		22 (3.7)
5 children		4 (0.7)
6 children		3 (0.3)
Total number of children missing		28 (4.7)
Age of children	23.2 (11.0) Range:0–59 years	

**TABLE 4:** Descriptive statistics for study variables.

***N* = 771**	**Possible range**	**Mean (SD)**
Attachment styles		
Anxiety	1–7	2.32 (1.49)
Avoidant	1–7	3.48 (1.92)
Psychological Adjustment		
Distress	0–10	5.55 (2.59)
Supportive Care Needs		
Health system/information needs	0–100	46.35 (33.63)
Patient care and support needs	0–100	35.27 (31.86)
Psychological Needs	0–100	51.18 (29.71)
Physical and daily living needs	0–100	40.12 (26.97)
Sexual needs	0–100	43.53 (33.10)

### Relationship Between Attachment Styles and Supportive Care Needs

In the first step of the analysis, a multiple linear regression model was used to explore the influence of the attachment styles (anxiety and avoidance) on each dimension of the SCNS-SF-34. Attachment anxiety was a significant determinant of all dimensions of the SCNS-SF-34, whereas attachment avoidance was not a significant determinant in our regression model (Table 5). For the *health system/information needs* dimension, attachment anxiety explained 6% (*R*^2^ = 0.06) of the variance, and it was a significant determinant (β = 5.45, *p* < 0.001). For the *patient care and support needs* dimension, attachment anxiety explained 7% (*R*^2^ = 0.07) of the variance, and attachment anxiety was a significant determinant (β = 1.31, *p* < 0.001). Over 10% (*R*^2^ = 0.10) of the variance in the *psychological needs* dimension could be explained by attachment anxiety and it was a significant determinant (β = 1.50, *p* < 0.001). Attachment anxiety explained 5% (*R*^2^ = 0.05) of the variance in the *physical and daily living needs* dimension and attachment anxiety was a significant determinant (β = 0.96, *p* < 0.001). For the *sexual needs* dimension, attachment anxiety explained 11% (*R*^2^ = 0.11) of the variance and attachment anxiety was a significant determinant (β = 1.74, *p* < 0.001).

**TABLE 5:** Multiple regression analysis of supportive care needs with anxious and avoidant attachment styles as determinants.

***Variables***	B*	**SE^†^**	**β^‡^**	***p*-value**	***R*^2^**
Health system/information needs					0.06
Intercept	32.80	2.85		<001	
Anxious	5.45	0.80	0.24	<**001**	
Avoidant	0.25	0.62	0.01	0.69	
Patient care and support needs					0.07
Intercept	19.16	2.69		<001	
Anxious	1.31	0.19	0.24	<**001**	
Avoidant	0.29	0.15	0.07	0.05	
Psychological needs					0.10
Intercept	34.15	2.47		<001	
Anxious	1.50	0.17	0.30	<**001**	
Avoidant	0.22	0.13	0.06	0.09	
Physical and daily living needs					0.05
Intercept	29.19	2.30		<001	
Anxious	0.96	0.16	0.21	<**001**	
Avoidant	0.15	0.13	0.04	0.24	
Sexual needs					0.11
Intercept	23.45	2.73		<001	
Anxious	1.74	0.19	0.31	<**001**	
Avoidant	0.28	0.15	0.07	0.06	

*p < 0.05. Bold values are significant.*

### Interaction Between Attachment Anxiety and Distress

**FIGURE 2:**
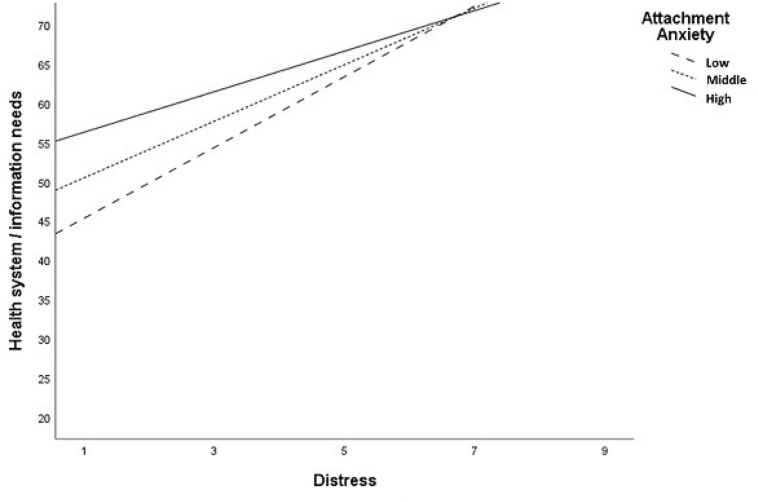
Significant interaction between attachment anxiety and distress amoung *Health system/information needs* domain.

**FIGURE 3:**
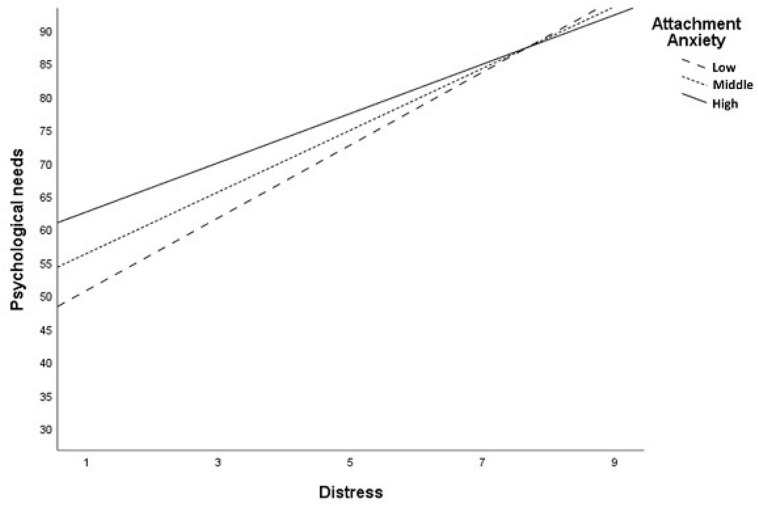
Significant interaction between attachment anxiety and distress among *Psychological needs* domain.

Based on conceptual considerations regarding the special impact of attachment anxiety under conditions of subjective distress, we assessed the influence of the interaction between attachment anxiety and distress on the dependent variables. The interaction effect was used as a moderator for all five need dimensions of the SCNS-SF-34 in this model. Taken together, distress as an additional determinant led to higher explanation of variance. Further, the interaction between attachment anxiety and distress became significant for the *health system/information needs and psychological needs* dimension. For the other dimensions the interaction was not significant. The results showed that distress moderates the effect and leads to higher unmet supportive care needs of the dimensions of *health system/information needs*, and *psychological needs*. These data are shown in Table 6 and in Figures 2,3.

**TABLE 6:** Moderation analysis with the interaction of attachment anxiety and distress.

**Variables**	**Coefficient**	**SE^†^**	**LLCI**	**ULCI**	***p*-value**	***R*^2^**
Health system/information needs						0.14
Constant	46.90	1.17	44.60	49.19	<0.001	
Attachment anxiety	4.59	0.79	3.04	6.12	**<0.001**	
Distress	3.60	0.45	2.72	4.49	**<0.001**	
Interaction term	−0.68	0.31	−1.29	−0.08	**0.02**	
Patient care and support needs						0.14
Constant	35.70	1.12	33.49	37.90	<0.001	
Attachment anxiety	4.60	0.79	3.04	6.16	**<0.001**	
Distress	3.19	0.43	2.35	4.04	**<0.001**	
Interaction term	−0.53	0.31	−1.15	0.09	0.09	
Psychological needs						0.27
Constant	51.70	0.96	49.82	53.57	<0.001	
Attachment anxiety	4.85	0.64	3.59	6.10	**<0.001**	
Distress	4.65	0.38	3.91	5.39	**<0.001**	
Interaction term	−0.63	0.25	−1.13	−0.13	**0.01**	
Physical and daily living needs						0.21
Constant	40.09	0.89	38.34	41.83	<0.001	
Attachment anxiety	2.36	0.60	1.18	3.53	**<0.001**	
Distress	4.34	0.35	3.64	5.03	**<0.001**	
Interaction term	0.04	0.24	−0.44	0.52	0.87	
Sexual needs						0.18
Constant	43.60	1.15	41.33	45.85	<0.001	
Attachment anxiety	5.99	0.83	4.37	7.61	**<0.001**	
Distress	3.46	0.43	2.61	4.30	**<0.001**	
Interaction term	−0.09	0.31	−0.70	0.53	0.78	

*p < 0.05. For a correct estimation of the influence of the single factors attachment anxiety and distress were centred. LLCI, Lower Level Confidence Interval; ULCI, Upper Level Confidence Interval. Bold values are significant.*

## Discussion

To our knowledge, this is the first study to address the possible determinants of attachment styles on the perception of supportive care needs among women with breast and gynecological cancer. In this sample of 771 cancer patients, we found that attachment styles, especially attachment anxiety, led to significantly higher perceived unmet needs in all supportive care need dimensions. In contrast, patients with attachment avoidance did not express higher unmet supportive care needs. We subsequently used a logistic path analytic model to better define the interaction between attachment anxiety and supportive care needs. Explained variance was higher when attachment anxiety, distress and their interaction were included as determinants in our model. We identified a significant interaction between attachment anxiety and distress within the *psychological needs*, *health system*, and *information needs* dimensions. On the other hand, for the dimensions *patient care, physical*, and *sexual needs* attachment anxiety led to a higher development of unmet supportive care needs independently of experienced distress.

Our findings are in line with the attachment theory, as anxiously attached individuals have a strong motive to turn to others in times of need. Anxiously attached individuals are at the same time likely to feel uncomfortable receiving support from others and can neither focus on nor express their needs during times of distress. In particular, they are experiencing increased fear of rejection or abandonment and are thus unable to ask and seek support (Bowlby, 1969). Interestingly, in our study, the assessed supportive care needs of the patients with attachment anxiety could, for the most part, be satisfied by medical care teams, psychosocial assistants or family members. It seems likely that patients with an anxious attachment style are not able to ask or seek for support. Due to this behavior their supportive care needs remain unsatisfied. These findings resonate with the theoretical model of Maunder and Hunter, which states that less effective help-seeking behavior is problematic for insecurely attached persons (high attachment anxiety and/or avoidance) (Maunder and Hunter, 2001; Graetz et al., 2013). Moreover, securely attached individuals (less attachment anxiety and/or avoidance) may be more likely to use active and positive coping strategies to overcome their cancer-related burdens, which are predictive for a positive psychological outcome in cancer patients. It seems that active coping mechanisms, such as planning, positive reframing, acceptance techniques and social support are positive strategies that may have the potential to support post-traumatic growth in cancer patients and reduce unmet needs (Schmidt et al., 2002, 2012; Romeo et al., 2017, 2019).

According to our findings, we assumed that patients with attachment anxiety suffer from higher unmet supportive care needs due to maladaptive coping strategies. Similarly, it has been shown that patients with a hepatitis C (Ciechanowski et al., 2002), cardiovascular diseases (McWilliams and Bailey, 2010) or chronic pain (McWilliams et al., 2000; McWilliams, 2017) and attachment anxiety tend to report physical symptoms that are not explained by their underlying illness. Furthermore, Ciechanowski et al. (2003) and Schroeter et al. (2015) found that ratings of insecure adult attachment are positively associated with depressive symptoms in patients with chronic pain.

In further studies it was shown that attachment anxiety interacts with higher physical and depression symptoms (Taylor et al., 2000; Kim et al., 2008; Smith et al., 2018). This could also be a possible explanation why patients with attachment anxiety may develop more unmet supportive care needs. Taylor et al. (2000) showed that patients with unexplained physical symptoms are more likely to have an insecure attachment style and psychiatric stress. This can be in line with our findings since distressed patients with attachment anxiety may suffer from higher unexplained somatic symptoms. In consequence, this may also lead to higher psychological burden and higher unmet supportive care needs.

In contrast, individuals with higher attachment avoidance develop a need for independence and self-sufficiency. This behavior might be a consequence of experiences of unresponsive parenting during childhood. Therefore, patients with attachment avoidance are uncomfortable getting close to others in times of need (Bowlby, 1969; Brandão et al., 2018). As a result, one can assume that support from others is not useful to overcome burden, even in cases of hazardous diseases such as cancer (Mikulincer et al., 2003). Attachment avoidance is also associated with a tendency to downplay threat and disease-related burden (Hunter et al., 2006). It seems likely that such attachment behavior led to disregardment of elevated unmet supportive care needs in our study.

Hamama-Raz and Solomon found that melanoma survivors with attachment anxiety experience increased distress compared to melanoma survivors with attachment avoidance (Hamama-Raz and Solomon, 2006). These findings are inconsistent with our data. Within our sample, we found that avoidantly attached patients did not differ in their distress score compared to patients with attachment anxiety. Moreover, the interaction effects of attachment anxiety and distress were not identified as significant determinants of all assessed supportive care needs suggesting that distress is not the only reason for unmet supportive care needs of patients with an insecure attachment style. This may be seen as consistent with the attachment theory, which postulates that attachment styles are internal models which are stable overtime, independent of external factors (Bowlby, 1969; Ainsworth et al., 2014). Therefore, it can be assumed that the effect of attachment anxiety on the development of supportive care needs is not significantly influenced by experienced distress. However, in our study, distress and attachment anxiety did interact with *psychological needs* and *health care needs*. A possible explanation of the interaction among *psychological needs* and *health care needs* is that the items of these two dimensions in the SCNS-SF-34 measured a similar experienced burden such as the DT. It seems that the items of both questionnaires are not selective enough. Both questionnaires measure burden in general and do not measure specific psychosocial aspects of experienced burden (Mehnert et al., 2006; Lehmann et al., 2012). For this reason, the interaction of attachment anxiety and distress led to higher unmet *psychological needs* in our study. Lehmann et al. (2012), in a validation study of the SCNS-SF-34, reported similar findings with the DT and the *psychological needs* dimension.

Taken together, we propose that insecure attachment styles, especially attachment anxiety, make it more likely that a patient will perceive a lack of support to address specific supportive care needs compared to patients with attachment security.

Additionally, attachment styles may constitute a risk factor resulting in poor well-being, independently of perceived distress among cancer patients.

Our exploratory study was based on a large sample of patients with breast cancer, gynecological cancer, or both. However, there are limitations in the sample selection and generalizability of this study. The lack of diversity in this sample is demonstrated by the participants being predominantly younger and highly distressed. In our sample, the mean distress score was 5.55, which is higher than reported in other studies reflecting a highly burdened cohort (Dabrowski et al., 2007; Ng et al., 2017). Therefore, a recruitment bias cannot be ruled out in our study. It is important to note that mainly women with breast cancer (87.0%) participated in our survey. Although this trend has been observed in other similar studies, further studies including other tumor entities, as well as male patients, are needed. For this reason, statements concerning other tumor entities and men cannot be made at this point. Additional studies will be needed to clarify whether additional factors (e.g., depression/anxiety symptoms, relationship issues and adverse disease experiences) have a role in the development of unmet supportive care needs.

In summary, our findings showed that individuals with attachment anxiety develop higher unmet supportive care needs independent of perceived distress. Thus, this group may be at greater risk of experiencing an impaired adjustment to their cancer diagnosis. Patients with attachment avoidance may not express increased unmet supportive care needs, while still suffering from high levels of distress. Therefore, clinicians should be aware that avoidant attachment behavior can impede the identification of patients in need of psycho-oncological services (Porter et al., 2012; Maunder and Hunter, 2016). Patients with an avoidant attachment are likely to decline help and patients with an anxious attachment are, at least partly, unable to seek and ask for the required support in times of need (Turner-Cobb et al., 2002; Brandão et al., 2018). An awareness of the influence of attachment styles, especially, attachment anxiety and avoidance on the supportive care needs of patients with cancer is necessary in clinical (psychosomatic) practice. Here, we propose that an attachment style questionnaire could be added to established distress tools assessing psycho-oncological support needs since highly distressed patients often decline help in a psycho-oncological screening and therefore do not receive support (Clover et al., 2015). The use of attachment style questionnaires might help to avoid adverse psycho-social consequences, which in turn may improve the somatic course of cancer treatment (e.g., *via* adherence to medications or treatment regimens) (Shorey and Snyder, 2006; Romeo et al., 2019). By such an approach, clinicians could better understand their patients’ needs and, therefore, more selectively offer the adequate psychosocial support that is most likely to satisfy the unmet supportive care needs of their patients.

## Data Availability Statement

The raw data supporting the conclusions of this article will be made available by the authors, without undue reservation.

## Ethics Statement

The studies involving human participants were reviewed and approved by Ethics committee of the University Hospital Tuebingen. The patients/participants provided their written informed consent to participate in this study.

## Author Contributions

JG: conception, data acquisition, data analysis, interpretation of the results, and writing of the manuscript. FJ: conception, and contributions to the writing of the manuscript, and interpretation of the results. JE: assistance in data acquisition, conception, and contributions to the writing of the manuscript. NS, JS-K, AS, AM-T, SB, and SZ: conception, contributions to the writing of the manuscript. LM: conception, assistance in data acquisition, and contributions to the writing of the manuscript. MT: conception, data analysis, interpretation of the results, and contributions to the writing of the manuscript. All authors contributed to the article and approved the submitted version.

## Conflict of Interest

The authors declare that the research was conducted in the absence of any commercial or financial relationships that could be construed as a potential conflict of interest.
